# Autoimmune Encephalitis in Latin America: A Critical Review

**DOI:** 10.3389/fneur.2020.606350

**Published:** 2021-01-21

**Authors:** Gabriel de Albuquerque Vasconcelos, Rodrigo Montenegro Barreira, Karmelita Emanuelle Nogueira Torres Antoniollo, Alina Maria Nuñez Pinheiro, Cíntia Fernandes Rodrigues Maia, Danyela Martins Bezerra Soares Alves, Paulo Ribeiro Nóbrega, Pedro Braga-Neto

**Affiliations:** ^1^Center of Health Sciences, Universidade Estadual do Ceará, Fortaleza, Brazil; ^2^Division of Neurology, Department of Clinical Medicine, Fortaleza, Brazil; ^3^Neurology Service, Hospital Geral de Fortaleza, Fortaleza, Brazil

**Keywords:** encephalitis, anti-N-methyl D-aspartate receptor encephalitis, neuroimmunology, autoimmune encephalitis, Latin America, autoimmune diseases

## Abstract

Autoimmune encephalitis is an increasingly recognized cause of encephalitis. The majority of case series report patients residing in developed countries in the northern hemisphere. The epidemiologic features of autoimmune encephalitis in Latin America are still unclear. The aim of the study was to perform a review of the clinical presentation of autoimmune encephalitis in Latin America and compare to world literature. References were identified by an in-depth literature search and selected on the basis of relevance to the topic and authors' judgment. We selected clinical studies and case reports published from 2007 to July, 2020 including patients from Latin American countries. Of the 379 patients included, the majority were cases of anti-NMDA receptor encephalitis (93.14%), followed by anti-VGKC-complex encephalitis (*N* = 17; 4.48%), anti-GAD encephalitis (*N* = 9; 2.37%), anti-AMPA receptor encephalitis (*N* = 1; 0.26%), anti-GABA receptor encephalitis (*N* = 1; 0. 26%), anti-mGluR5 encephalitis (*N* = 1; 0. 26%), and anti-mGluR1 encephalitis (*N* = 1; 0. 26%). Reported cases of Anti-NMDA encephalitis in Latin-America had a very slight female predominance, lower prevalence of associated tumors and a lower incidence of extreme delta brush on electroencephalogram. Autoimmune encephalitis is possibly underdiagnosed in underdeveloped countries. Its outcome after treatment, however, appears to be similarly favorable in Latin American patients as has been reported in developed countries based on available case reports and case series. Regional specificities in the manifestation of autoimmune encephalitis could be related to epidemiologic factors, such as the presence of different triggers and different genetic and immunologic background, that need to be studied by future research.

## Introduction

There has been an increasing worldwide awareness of autoimmune encephalitis (1), which is characterized by the production of antibodies against neuronal cell surface and synaptic molecules, causing different clinical manifestations depending on the antibody produced (2, 3). Diagnostic criteria for possible autoimmune encephalitis are based on clinical presentation, magnetic resonance imaging (MRI) and electroencephalography (EEG) results, and exclusion of other pathologies. Definitive diagnosis is established by demonstration of specific antibodies in the blood and/or cerebrospinal fluid, although the absence of antibodies does not rule out an autoimmune etiology (4).

The majority of studies on autoimmune encephalitis have been carried out in developed countries, whose sociodemographic variables differ from those of developing countries. Most studies in Latin America have small samples, and some series report on syndromes caused by different antibodies, leading to heterogeneity. These limitations, combined with the ethnic background of the Latin American population, of similar colonial origin and closely related indigenous population, justifies the conduction of a literature review to investigate particularities in the presentation of autoimmune encephalitis in this population. Therefore, the present study aims to describe clinical manifestations of autoimmune encephalitis in Latin America and to compare the results with what is reported in world literature.

## Methods

### Literature Review

**Figure 1:**
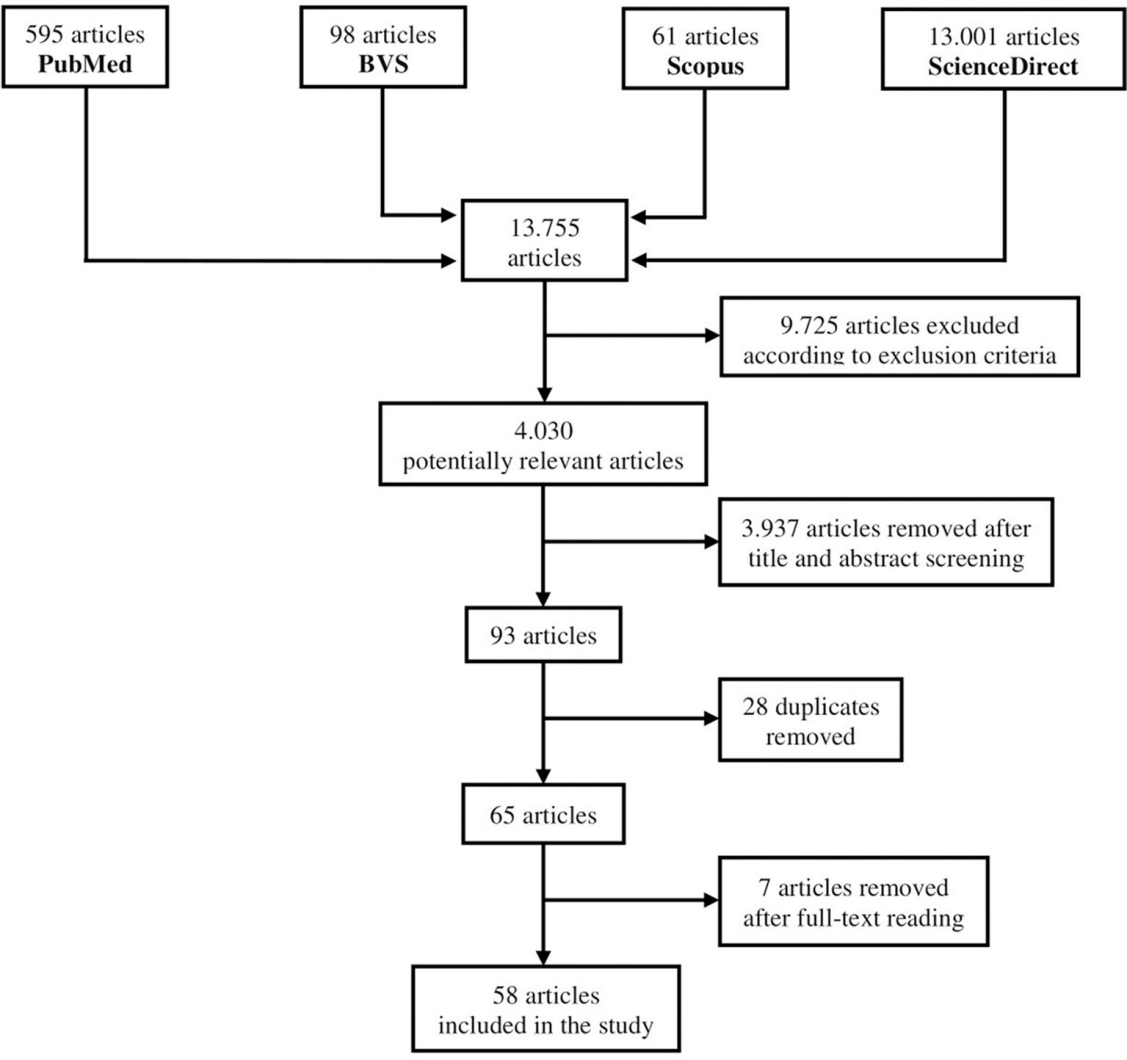
Literature review flowchart.

We performed a narrative literature review. References were identified through searches of PubMed, Scopus, ScienceDirect, and BVS with search terms according to Figure 1. Articles dating from January, 2007 to July, 2020 were included. The final reference list was generated based on relevance to the scope of this review. The search yielded 13,755 articles. Selection was based on the review of the title and abstract of the articles.

Inclusion criteria were: case series and case reports on autoimmune encephalitis associated with antibodies against cell surface and synaptic proteins in Latin American patients, published from 2007 to 2020.

Exclusion criteria were: articles published in languages other than English, Portuguese, or Spanish, articles published before 2007, or articles with only an abstract available. After applying the exclusion criteria, 9,725 articles were excluded, leaving 4,030 articles for further analysis. One article was not included due to impossibility of accessing study's data.

After title and abstract screening, 93 articles remained, and after duplicates were removed, 65 articles were selected for full-text reading. After full-text reading, seven articles were excluded for having a content that diverged from the objectives of this review. These steps are summarized in the flow-chart (Figure 1).

### Data Analysis and Presentation

The following criteria were used to describe the variables in Table 1. Sample group was composed only of patients with a diagnosis confirmed by the finding of antineuronal surface antibodies. Mean age and standard deviation, when not described by the study, were calculated, except in articles that did not reveal the age of each patient and only reported mean age; in these cases, standard deviation was considered not reported. The percentage of female patients was not reported in only one study.

**Table 1:** Autoimmune encephalitis reports in Latin America by antibody type.

**Antibody**	**Number of patients**	**Age (mean)**	**Female (%)**	**Movement disorders (%)**	**Seizures (%)**	**Cognitive decline (%)**	**CSF pleocytosis (%)**	**Hyperintensity on MRI (%)**
NMDAR	353	21.6	50.15%	52.9%	55.52%	28.70%	53.51%	29.22%
1LGI1	14	62.21	58.33%	85.71%	78.57%	100%	50%	71.43%
GAD	9	42.89	77.78%	33.33%	55.56%	44.44%	0%	77.78%
VGKC	3	71.33	33.33%	100%	0%	66.67%	NR	100%
AMPAR	1	67	100%	0%	0%	100%	0%	0%
GABAA	1	66	0%	0%	100%	0%	0%	100%
MGLUR1	1	22	100%	0%	0%	100%	100%	0%
MGLUR5	1	68	100%	0%	0%	100%	0%	0%
Total	383	24.34	51.08%	52.29%	55.8%	32.20%	50.38%	35.87%

*NR, Not Reported; MRI, Magnetic resonance imaging; CSF, Cerebrospinal Fluid*.

Frequency of symptoms such as behavior changes, cognitive decline, movement disorders, seizures, dysautonomia and hyponatremia were expressed as percentages of the total number of patients whose symptoms were described.

If results of EEG, CSF, or MRI examinations were not reported, the variables “EEG,” “CSF pleocytosis” and “hyperintensity on MRI” were considered not reported. If the test result was abnormal, but not specified, the variable was considered described, but not specified. The percentage of patients diagnosed with a tumor was described for papers that reported a systematic search for a tumor.

## Results

The studies reviewed amounted to a total of 383 patients with a confirmed diagnosis of autoimmune encephalitis by detection of antibodies in the blood and/or cerebrospinal fluid. Most patients were diagnosed with anti-NMDA receptor encephalitis (*N* = 353; 93.14%), followed by anti-VGKC-complex encephalitis (*N* = 17; 4.48%), anti-GAD associated encephalitis (*N* = 9; 2.37%), anti-AMPA receptor encephalitis (*N* = 1; 0.26%), anti-GABA receptor encephalitis (*N* = 1; 0.26%), anti-mGluR5 encephalitis (*N* = 1; 0.26%), and anti-mGluR1 encephalitis (*N* = 1; 0.26%). In patients whose age was reported 60.78% were under 18 years of age.

Patients presented mostly with encephalopathy associated with behavior changes (85.51%), seizures (55.8%), movement disorders (52.29%) and cognitive decline (32.20%). Most of them retrospectively fulfilled Graus criteria for possible autoimmune encephalitis (4). Dysautonomia (28.1%) and hyponatremia (2.87%) were also observed in some patients. EEG was abnormal in most patients (89.07%) ranging from slow baseline activity to epileptiform discharges and status epilepticus. Hyperintensity on MRI was observed in 35.87%.

Treatment was performed in most patients with methylprednisolone (95.3%), immunoglobulin (60.2%) and/or plasmapheresis (36.8%). Second-line therapy was administered in 44.4% with rituximab (27%) or cyclophosphamide (26%). The majority of patients improved after immunotherapy, and in most cases where final outcomes were available they were discharged with mild to moderate disability. The modified ranking scale (mRS) score at last documented follow-up assessment was reported for 43 patients in 4 studies. Of those, 23 patients had an mRS score of 0, 12 patients had an mRS score of 1, two patients had an mRS score of 2, three patients had an mRS score of 3, two patients had an mRS score of 4, and one patient had an mRS score of 6 (3, 5–7). An mRS ≤ 2 has been achieved in 86% of patients. Time from disease onset at last follow-up was variable, ranging from 3 to 116 months.

### Anti-NMDA Receptor Encephalitis

Patients with anti-NMDA receptor encephalitis (ANMDARE) were mostly children, adolescents (73.81% under 18 years of age) and young adults, with a mean age ranging from 5 to 44 years, and a slight female predominance (*N* = 172; 50.15%) (Supplementary Table). Articles that reported this diagnosis were from Brazil, Argentina, Mexico, Peru, Costa Rica, Ecuador, Venezuela and Colombia. The test to find antibodies was specified for 326 patients (92.35%). Antibodies were found only in cerebrospinal fluid in most patients (*N* = 189; 57.97%), in cerebrospinal fluid and serum (*N* = 136; 41.72%), or only in serum (*N* = 1; 0.3%).

In patients whose initial clinical features were described (*N* = 152), the majority had some psychiatric symptom at initial presentation (*N* = 102; 67.11%), mainly psychomotor agitation and irritability. A systematic search for tumor was reported for 252 patients (71.39%). Sixteen were diagnosed with ovarian teratoma (6.35%), one was diagnosed with serous ovarian cystadenoma (0.4%), one was diagnosed with papillary thyroid carcinoma (0.4%), and one was diagnosed with a complex ovarian cyst (0.4%), all female. Cases associated with known triggers were due to Epstein-Barr virus infection (1 case in Brazil), chikungunya virus infection (1 case in Brazil), mycoplasma infection (5 cases in Argentina), human herpes virus 7 infection (5 cases in Mexico), human herpes virus 6 infection (1 case in Mexico), and Varicella-zoster infection (1 case in Costa Rica and 2 cases in Chile) (7–12). Transfer to Intensive Care Unit (ICU) was reported in only 9.35% of patients (*N* = 33).

For studies which specified the results of CSF examination, pleocytosis was the most frequent finding (*N* = 61; 53.51%), sometimes accompanied by elevated CSF protein or oligoclonal bands. For some patients, the performance of an MRI examination was not reported (*N* = 79; 22.38%), or, if reported, the abnormal results were not specified (*N* = 120; 43.8%). Among patients whose MR results were known, 70.78% were normal, and hyperintensity was the most important abnormal finding (*N* = 45; 29.22%) (Table 1), with hyperintensity in the mesial temporal lobe prevailing. EEG was performed in 348 patients (98.58%). Of these, results were abnormal in 313 (89.94%). EDB was an uncommon finding (*N* = 24; 12.06%) and findings such as irregular theta and delta waves, diffuse slowing, and epileptiform activity prevailed. Abnormalities were not specified for 149 patients (42.82%).

### Anti-VGKC Complex Encephalitis

Anti-VGKC complex antibodies were found in serum in 17 patients in Brazil, Peru Argentina, and Chile, of which 14 were described as anti-LGI1 positive (Supplementary Table). No case of anti-CASPR2 encephalitis was reported in the sample. Patients were predominantly female (*N* = 8; 53.33%) and elderly, with a mean age ranging from 55 to 74 years. In addition to psychiatric symptoms (*N* = 11; 64.71%), and seizures (*N* = 11; 64.71%), cognitive decline associated with memory impairment (*N* = 17; 100%) were important clinical findings. Faciobrachial dystonic seizures were reported in 11 patients (64.71%). None of the patients were reported to have been transferred to intensive care and only one had autonomic dysfunction. Despite the systematic search for a tumor in six patients, neither neoplasia nor any association with an infectious trigger was detected. The frequency of hyponatremia was relatively low (*N* = 9; 52.94%%). EEG was altered in 70.59% of cases, generally with slow activity. Hyperintensities on MRI (*N* = 13; 76.47%) were seen predominantly in mesial temporal lobes and basal ganglia, mainly in putamen and caudate.

### Anti-GAD Encephalitis

Patients with anti-GAD-antibody associated encephalitis (*N* = 9) were reported only in Brazil. They were predominantly young women (*N* = 7; 77.78%), with a mean age ranging from 19 to 58.5 years, with all antibodies obtained from serum samples (Supplementary Table). Epileptic seizures, mainly tonic-clonic and myoclonic seizures, were present in 55.56% of the patients. One patient had opercular myoclonic-anarthric status epilepticus (*N* = 1; 11.11%). Only for three patients (33.33%) a search for a tumor was described, which was negative. Only one patient (11.11%) in the sample had autoimmune diabetes mellitus. EEG examination was reported for some patients (*N* = 6; 66.67%), and all these patients had some abnormality, mainly epileptiform paroxysms. Five patients (55.56%) underwent CSF examination and results were normal for all patients. Hyperintensity on MRI, predominantly frontotemporal and hippocampal, were found in seven patients (77.78%). In two patients (20%) MRI revealed cerebellar atrophy, clinically manifested by cerebellar ataxia with postural instability, impairment of horizontal saccades, and nystagmus (13).

### Others

The only case of anti-AMPA receptor encephalitis was reported in Brazil and was associated with small-cell lung carcinoma (3). There was only one case of anti-GABA receptor encephalitis, which was described in a Chilean study (14). The only patient diagnosed with anti-mGluR5 encephalitis was also described in a Chilean study. This patient concomitantly had Hodgkin lymphoma (Ophelia syndrome) (15). In addition, in Guadaloupe, a case of anti-mGluR1 encephalitis that presented with acute cerebellar ataxia was associated with a possible trigger by dengue virus infection (16).

## Discussion

The diagnosis of autoimmune encephalitis is complex due to several factors, such as lack of awareness by medical professionals, difficulty in analyzing body fluids for the detection of antibodies, variable clinical phenotype, and manifestations similar to those of other diseases. There are considerably fewer reports of autoimmune encephalitis in Latin American patients compared to those from developed countries.

One of the studies in this review reported an estimated incidence of autoimmune encephalitis in a region of Brazil of 0.16/100,000 person-years (3), significantly less than the incidence described by a study in Minnesota, United States of America, of 0.8/100,000 person-years (17). This difference could be explained by underdiagnosis. Latin American countries have low average income and variable levels of healthcare system quality and a lot of people in these countries live in isolated areas with difficult access to tertiary care. Availability of technology dependent diagnostic methods, such as MRI, EEG and advanced CSF analysis is also scarce in these regions. CSF and blood samples for suspect cases of autoimmune encephalitis usually have to be sent to Europe (3) or the United States for analysis, which is usually done under research grants, and these grants are limited due to inadequate funding. We believe these factors are responsible for underdiagnosis, which accounts for the small number of cases found in our literature review. The development and incorporation of techniques for neuronal cell surface antibody research by local laboratories could improve diagnostic yield in low income countries.

According to four studies included in this review, which add up to 43 patients, scores on the modified ranking scale (mRs) at the last evaluation during follow-up revealed that what has been considered as a “good outcome” (mRs ≤ 2) in previous reports has been achieved in the majority of patients (*N* = 37; 86.05%) (3, 5–7). This is consistent with larger data from international literature where an mRs ≤ 2 was achieved in 78.64% of patients (18). The notorious therapeutic response, associated with the possibility of underdiagnosis, reinforces the need for greater awareness of the disease by specialists working in underdeveloped countries.

As far as we know, this is the first study to review the clinical presentation of autoimmune encephalitis associated with anti-neuronal antibodies in Latin America. It is therefore necessary to describe basic characteristics of the encephalitis subtypes studied, based on literature reviews, and compare these characteristics with the results obtained.

### Anti-NMDA Receptor Encephalitis

ANMDARE often starts with a prodromal phase, followed by psychiatric and cognitive symptoms, movement disorders, seizures, autonomic instability, and central hypoventilation (2, 19). It predominantly affects young women, with a mean age of 21 years (19).

This review included a significant number of children (73.81% under 18 years of age) with 14 articles composed exclusively of pediatric patients (7–9, 11, 12, 20–28). In a large series of anti-NMDAR Encephalitis 37% of patients were children (18). The discrepancy in the percentage of children might be due to a publication bias, as ANMDARE in children tends to be dramatic in presentation and response to therapy. Also, the differential diagnosis in older adults with possible autoimmune encephalitis is broader and includes a large number of neurodegenerative disorders, whereas in children the primary differential diagnosis is viral encephalitis, which is easier to exclude. This could have led to a higher proportion of diagnoses in children in a setting of limited technological resources. Finally, it is also possible that a higher number of post-infectious cases as opposed to paraneoplastic cases could explain this higher proportion of children (2).

There was a very slight female predominance (50.15%), whereas in literature a substantial female predominance (81%) is reported (18). This finding is possibly related to the large proportion of children, in which female preponderance is less marked. It could also be related to a lesser proportion of paraneoplastic etiologies, which are more common in women (18).

In an international series of 577 cases of anti-NMDA encephalitis, 220 (38%) had an underlying tumor, of which 213 were women (18). Most prevalent was ovarian teratoma (94%), but also extraovarian teratomas (2%), lung tumors (1%), breast tumors (1%), testicular tumors (1%), ovarian carcinoma (0.5%), thymic carcinomas (0.5%), and pancreatic cancer (0.5%) were present (18). In the present study, there was a small number of patients diagnosed with a tumor (*N* = 19; 7.54%) (Table 1), despite the systematic search in most cases. It is possible that this result is a consequence of the large number of pediatric cases included in the study, since in women with ANMDARE tumors are usually diagnosed after the age of 18 (2). Another hypothesis is that a greater number of infectious triggers in Latin American patients could reduce the proportion of cases with a paraneoplastic etiology. A variation in prevalence of underlying tumors has also been observed international case series, with a higher percentage in Asians (45%) and African-Americans (48%) compared to Caucasians (31%) and Hispanics (27%) (18). However, these results could be partly due to limited resources for cancer screening.

Also, 9.35% of patients were reported to be admitted to intensive care units compared to 70% in literature (19). Since reasons for intensive care admission, such as hypoventilation and dysautonomia were frequent, this discrepancy could be explained by failure to report this information or the limited availability of ICU beds in Latin American health services.

The results of MRI and CSF examinations are in agreement with what is reported in literature. For EEG examination the percentage of patients in the sample with EDB (12.06%) was approximately half of that described in literature (30%) (29). This could imply a lack of awareness of EDB by neurophysiologists in Latin America.

### Anti-VGKC Encephalitis

Antibodies against the voltage-dependent potassium channel (VGKC) were identified in 1995 in patients with neuromyotonia (30). It has been subsequently demonstrated that these syndromes were caused by antibodies against leucine-rich glioma-inactivated 1 (LGI1) and contactin-associated protein-like 2 (Caspr2), proteins related to VGKC (30). In this study, the subgroup of antibodies was not specified for 2 patients, while all others had anti-LGI1 antibodies (31, 32).

The clinical phenotype of anti -LGI1 encephalitis is usually manifested as cognitive impairment, seizures, faciobrachial dystonic seizures (FBDS), and hyponatremia (30). Anti-LGI1 encephalitis is difficult to diagnose, as it can cause rapidly progressive dementia (30), as observed in two cases in the sample (32). Another possible mistake associated with anti-LGI1 encephalitis is the frequent confusion of faciobrachial dystonic seizures with diverse movement disorders and focal seizures. In a study of three patients with anti-LGI1 encephalitis the presence of faciobrachial dystonic seizures was not specified, despite the description of nonspecific seizures in 100% of cases, possibly due to difficulty in distinguishing these two findings (5).

Results of CSF, laboratory, and EEG examinations were consistent with what is reported in the literature. However, hypersignal on MRI has been reported in 25% to 30% of patients in the acute stage of the disease (30), while in this review 76.47% of patients showed hyperintensity on MRI. Such divergent findings could be explained by the small sample.

### Others

Clinical presentation was consistent with what is reported in literature for patients with anti-GAD, anti-AMPA receptor, anti-GABA, anti-mGluR5, and anti-mGluR1 antibodies. Anti-GAD encephalitis may present with limbic encephalitis, ataxia, and refractory seizures (2). Anti-AMPA receptor encephalitis involves cognitive and psychiatric symptoms, sometimes associated with cancer (1). In the reported case, the patient had small-cell lung carcinoma (3). Anti-GABA receptor encephalitis causes cognitive impairment and behavior changes associated with seizures (1). The only reported case had refractory status epilepticus and psychiatric symptoms (14). Anti-mGluR5 encephalitis is characterized by psychiatric and cognitive symptoms and may be associated with Hodgkin lymphoma, composing the Ophelia Syndrome, as in the case reported (15). The main manifestation of anti-mGluR1 encephalitis is cerebellar ataxia, often (60%) related to Hodgkin lymphoma (2).

### Limitations

Unfortunately, proper epidemiological conclusions cannot be drawn from this review, as comprehensive epidemiological studies in Latin America were not found. There is a clear publication bias in the analysis of the studies reported here. These studies were also very heterogeneous in terms of sample and variables described. Most studies had very small samples and for all encephalitis except anti-NMDAR the total number of patients was very small to draw strong conclusions. Larger epidemiological studies in Latin America are needed to improve our knowledge in this field.

## Conclusions

Autoimmune encephalitis is a disease that is still possibly underdiagnosed in underdeveloped countries, such as those in Latin America. Its outcome after treatment, however, appears to be similarly favorable in Latin American patients as has been reported for patients in developed countries. Some particularities were found in reported cases of AE in these patients. In anti-NMDAR encephalitis there was a very slight female predominance, a lower incidence of associated tumors (possibly explained by a large number of pediatric cases, greater proportion of infectious triggers, or lower availability of resources for investigation), lower reporting of the need for intensive care, and lower percentage of patients with EDB on EEG in the cases that have been reported. In anti-LGI1 encephalitis there was a higher frequency of hypersignal on MRI, which is questionable, considering the small sample. The characteristics of other autoimmune encephalitis subtypes were very similar to what is reported in literature. This review should raise the awareness of clinicians working in underdeveloped countries to the possibility of autoimmune encephalitis and prompt the realization of larger studies to investigate particularities of this condition in these countries, such as the occurrence of previously unknown infectious triggers.

## Author Contributions

GV, RB, KA, PN, and PB-N: conception and design of the work. GV: literature search. GV, PN, and PB-N: acquisition, analysis, or interpretation of data for the work. GV, RB, KA, AP, CM, DA, PN, and PB-N: drafting the work. All authors were involved in critical revision of the manuscript for important intellectual content.

## Conflict of Interest

The authors declare that the research was conducted in the absence of any commercial or financial relationships that could be construed as a potential conflict of interest.
