# Supplementary material for: Autoimmune Encephalitis in Latin America: A Critical Review
Source: Front Neurol. 2021 Jan 21;11:606350. doi: 10.3389/fneur.2020.606350 (PMC7859257; doi:10.3389/fneur.2020.606350)
Supplement: Supplementary file 1 [file Table_1.docx]

Supplementary Table - Autoimmune encephalitis reports in Latin America. (3,5-16,20-28,33-66)

| **Reference/Sample** | **Antibody** | **Age mean** ± **SD** | **Sex (% women)** | **Extreme Delta Brush (%)** | **CSF pleocytosis (%)** | **Hyperintensity on MRI (%)** | **Tumor (%)** | **Seizures (%)** | **Psychiatric symptoms at presentation (%)** | **Movement disorders (%)** | **Dysautonomia (%)** |
| --- | --- | --- | --- | --- | --- | --- | --- | --- | --- | --- | --- |
| Nóbrega et al, 2019(3)/n=10 | NMDA | 16,4 ± 7,6 | NR | 20% | 50% | 20% | 0% | 60% | NR | 90% | 60% |
| Melamud et al, 2018(5)/n=4 | NMDA | 17,75 ±  5,44 | 100% | 0% | 100% | 50% | 0% | 100% | NR | 100% (NS) | 50% |
| Da Silva- Júnior et al, 2014(6)/n = 3 | NMDA | 24 ± 7,89 | 100% | 67% | 100% | 33% | NR (67%), 0% (33%) | 100% | 100% | 67% | NR |
| Chavez-Castillo et al, 2020(7)/n = 31 | NMDA | 10 ± NR | 35,5% | 9,68% | 54,94% | NR | 0% | 100% | 51,61% | NR | NR |
| Danieli et al, 2017(8)/n = 1 | NMDA | 6 ± 0 | 0% | 0% | 100% | 0% | 0% | 100% | 100% | 100% | 100% |
| Pérez et al, 2013(9)/n = 11 | NMDA | 6,9 ± NR | 64% | 0% | 54% | 27% | 0% | 81% | 27% | 54% | 100% |
| SOLÍS et al, 2016 (10)/n = 1 | NMDA | 29 ± 0 | 100% | 0% | 100% | 100% | 100% | 100% | 0% | 100% | 100% |
| Erazo et al, 2016 (11)/n = 13 | NMDA | 7,08 ± 4,65 | 30,77% | 0% | 7,69% | 0% | 0% | 76,92% | NR | 100% | 38,46% |
| Nóbrega et al, 2020 (12)/n = 1 | NMDA | 5 ± 0 | 0% | 100% | 100% | 0% | 0% | 100% | 0% | 100% | 100% |
| do Valle et al, 2019(20)/n = 9 | NMDA | 5 ± 5,1 | 67% | 0% | NR | 11% | 0% | 78% | 11% | 67% | 11% |
| Bravo-Oro et al., 2015 (21)/n = 1 | NMDA | 6 ± 0 | 100% | 0% | 100% | 100% | 0% | 100% | 0% | 100% | NR |
| Gonzalez-Latapi et al, 2014 (22)/n = 1 | NMDA | 16 ± 0 | 0% | 0% | 100% | 0% | 0% | 100% | 0% | 100% | NR |
| Bravo-Oro et al, 2013 (23)/n = 6 | NMDA | 5,1 ± 3,5 | 66,67% | 17% | 33% | 50% | 0% | 83% | NR | 100% | 67% |
| Borlot F et al, 2012 (24)/n = 3 | NMDA | 5 ± 3,56 | 66,67% | 0% | 33% | 0% | 0% | 67% | 67% | 100% | 33% |
| Benjumea-Cuartas et al, 2017 (25)/n = 1 | NMDA | 7 ± 0 | 0% | 0% | 100% | 0% | 0% | 100% | 0% | 100% | NR |
| Gómez et al, 2017 (26)/n = 1 | NMDA | 5 ± 0 | 0% | 0% | 0% | 0% | 0% | 100% | 100% | 100% | NR |
| Passareli et al, 2016 (27)/n = 1 | NMDA | 12 ± 0 | 0% | 100% | 100% | NR | NR | NR | 0% | 100% | NR |
| Rangel-Guerra et al, 2015 (28)/n = 1 | NMDA | 15 ± 0 | 100% | 0% | 0% | 0% | 0% | 100% | 100% | NR | NR |
| Martínez et al, 2019 (36)/n = 2 | NMDA | 22 ± 5,66 | 0% | 50% | 0% | 0% | NR | 100% | 100% | NR | NR |
| Palomino-Lescano, et al, 2019 (37)/n = 4 | NMDA | 29 ± 17,9 | 50% | 50% | 50% | 25% | 0% | 100% | 25% | 75% | 75% |
| Bustos et al, 2017 (39)/n = 2 | NMDA | 17,5 ± 5,5 | 100% | 0% | 50% | 0% | 0% | 100% | 50% | 50% | 100% |
| Kerik-Rotenberg et al, 2020 (40)/n = 33 | NMDA | 26,7 ± 9,2 | 45,50% | 18% | NR | 21% | 18,10% | NR | NR | 100% (NS) | NR |
| Restrepo-Martínez et al, 2020 (41)/n = 14 | NMDA | 24.5 ± 5.95 | 35.7 % | 100% (NS) | 100% (NS) | 100% (NS) | 7,14% | 71,40% | 78,60% | 100% (NS) | 35,7 |
| Restrepo-Martínez et al, 2020(43)/n = 3 | NMDA | 32,67 ± 1,16 | 33% | 0% | 33% | 0% | NR (33%), 0% (67%) | 67% | 100% | NR | NR |
| Reyna-Villasmil, et al, 2017 (44)/n = 1 | NMDA | 23 ± 0 | 100% | 0% | 100% | 0% | 100% | 100% | 100% | NR | NR |
| Cané et al, 2019(45)/n = 1 | NMDA | 23 ± 0 | 100% | 0% | 0% | 0% | 100% | NR | 100% | 100% | 100% |
| Martínez et al, 2012 (46)/n = 1 | NMDA | 25 ± 0 | 100% | 0% | 100% | 0% | 100% | NR | 100% | 100% | 100% |
| Jimenez-Zarazúa et al, 2019 (47)/n = 1 | NMDA | 34 ± 0 | 0% | 0% | 100% | 0% | 0% | 100% | 0% | 100% | NR |
| **Reference/Sample** | **Antibody** | **Age mean** ± **SD** | **Sex (% women)** | **Extreme Delta Brush (%)** | **CSF pleocytosis (%)** | **Hyperintensity on MRI (%)** | **Tumor (%)** | **Seizures (%)** | **Psychiatric symptoms at presentation (%)** | **Movement disorders (%)** | **Dysautonomia (%)** |
| Espinola-Nadurille et al, 2018 (49)/n = 48 | NMDA | 26.5 ± 9 | 50% | 100% (NS) | 100% (NS) | 100% (NS) | NR | 79,20% | NR | 37,5% | 50% |
| Soares et al, 2013 (50)/n = 1 | NMDA | 39 ± 0 | 100% | 0% | 100% | 0% | 0% | NR | 100% | 100% | 100% |
| Solis et al, 2020 (51)/n = 1 | NMDA | 22 ± 0 | 100% | 0% | 0% | 100% | 0% | 0% | 100% | NR | 100% |
| Espinola-Nadurille et al, 2019 (52)/n = 58 | NMDA | 25,9 ± 8,6 | 51% | 100% (NS) | 100% (NS) | 100% (NS) | 12,10% | 78% | NR | 100% (NS) | 48% |
| Martinez et al, 2018 (53)/n = 1 | NMDA | 32 ± 0 | 0% | 0% | 100% | 100% | 0% | 100% | 100% | NR | 100% |
| Monteiro et al, 2015 (54)/n = 1 | NMDA | 30 ± 0 | 0% | 0% | 100% | 0% | 0% | NR | 100% | 100% | 100% |
| Ramirez-Bermudez et al, 2020 (55)/n = 1 | NMDA | 44 ± 0 | 100% | NR | NR | NR | NR | 100% | 100% | 100% | 100% |
| Simabukuro et al, 2014(56)/n = 1 | NMDA | 18 ± 0 | 0% | 0% | 100% | 0% | 0% | 100% | 100% | 100% | 100% |
| Espinola-Nadurille et al, 2018 (57)/n = 29 | NMDA | 26,1 ± 9 | 55% | 100% (NS) | 100% (NS) | 65,50% | 0% | NR | NR | NR | NR |
| Bayliss et al, 2019 (58)/n = 1 | NMDA | 24 ± 0 | 100% | NR | NR | 0% | 100% | NR | 100% | NR | NR |
| Simabukuro et al, 2015(59)/n = 1 | NMDA | 34 ± 0 | 100% | 0% | 100% | 0% | 0% | 100% | 100% | 100% | 100% |
| Ávila et al, 2020 (60)/n = 45 | NMDA | 25,9 ± NR | 55,56% | 11,11% | NR | NR | NR | NR | 93,3% | NR | NR |
| Delgado-García et al, 2018 (61)/n = 1 | NMDA | 21 ± 0 | 0% | NR | NR | NR | NR | 100% | 0% | NR | NR |
| Pérez-Gilabert, 2017(63)/n = 1 | NMDA | 22 ± 0 | 0% | 0% | 100% | 0% | 0% | 100% | 100% | NR | 100% |
| Lozano-Cuervo et al, 2020 (64)/n = 1 | NMDA | 21 ± 0 | 0% | 0% | 100% | 100% | 0% | 0% | 100% | NR | NR |
| Da Silva et al, 2015 (66)/n = 1 | NMDA | 24 ± 0 | 0% | NR | 100% | 100% | 0% | NR | 100% | NR | NR |
| Nóbrega et al, 2019 (3)/n = 2 | LGI 1 | 74 ± 1,41 | NR | NR | 50% | 50% | 0% | NR | NR | 100% (FBDS) | NR |
| Melamud et al, 2018 (5)/n = 3 | LGI 1 | 60,67 ± 9,29 | 67% | NR | 67% | 100% | 0% | 100% | 100% | NR | 33% |
| Ibarra et al, 2015 (33)/n = 1 | LGI 1 | 58 ± 0 | 100% | NR | 0% | 100% | 0% | 100% | 100% | 100% (FBDS) | NR |
| Uribe-San-Martín et al, 2020 (34)/n = 6 | LGI 1 | 55,83 ± 10,92 | 50% | NR | NR | 50% | 0% | 100% | 0% | 66,67% | NR |
| Simabukuro et al, 2016 (42)/n = 2 | LGI 1 | 74 ± 2,83 | 50% | NR | 50% | 100% | NR | 50% | 50% | 100%(FBDS) | NR |
| Otárula et al, 2015 (31)/n = 2 | VGKC | 71 ± 7,07 | 0% | NR | NR | 100% | NR | NR | NR | 100% (FBDS) | NR |
| Alvarez et al, 2019 (32)/n = 1 | VGKC | 72 ± 0 | 100% | NR | NR | 100% | NR | NR | 100% | NR | NR |
| Vale, et al, 2015 (13)/n = 2 | GAD | 58,5 ± 10,6 | 0% | NR | 0% | 0% | 0% | NR | NR | NR | NR |
| Sairaiva et al, 2015 (35)/n = 1 | GAD | 54 ± 0 | 100% | NR | 0% | 100% | NR | NR | 100% | NR | NR |
| Nóbrega-Jr et al, 2018 (38)/n = 3 | GAD | 41 ± NR | 100% | NR | NR | 100% | NR | 67% | NR | NR | NR |
| Mendes et al, 2015 (48)/n = 1 | GAD | 19 ± 0 | 100% | NR | 0% | 100% | NR | 100% | 100% | NR | NR |
| Alencar et al, 2017 (62)/n = 1 | GAD | 50 ± 0 | 100% | NR | NR | 100% | NR | 100% | 100% | NR | 100%­ |
| Monnerat et al, 2013 (65)/n = 1 | GAD | 23 ± 0 | 100% | NR | 0% | 100% | 0% | 100% | NR | 100% | NR |
| **Reference/Sample** | **Antibody** | **Age mean** ± **SD** | **Sex (% women)** | **Extreme Delta Brush (%)** | **CSF pleocytosis (%)** | **Hyperintensity on MRI (%)** | **Tumor (%)** | **Seizures (%)** | **Psychiatric symptoms at presentation (%)** | **Movement disorders (%)** | **Dysautonomia (%)** |
| Nóbrega et al, 2019(3)/n=1 | AMPAR | 67 ± 0 | 100% | 0% | 0% | 0% | 100% | NR | 100% | NR | nr |
| González et al, 2016(14)/n=1 | GABAA | 66 ± 0 | 100% | 0% | 0% | 100% | 0% | 100% | 100% | NR | NR |
| Guevara et al, 2018(15)/n=1 | MGLUR5 | 68 ± 0 | 0% | 0% | 0% | 0% | 100% | NR | 100% | NR | NR |
| Chaumont et al, 2019(16)/n=1 | MGLUR1 | 22 ± 0 | 100% | 0% | 100% | 0% | 0% | NR | 100% | NR | NR |

SD = standard deviation CSF = cerebrospinal fluid. MRI = magnetic resonance imaging. NR = not reported. NS = described, but not specified. N/A = not applicable. FBDS = faciobrachial dystonic seizures.

**REFERENCE**

1. Dalmau J, Graus F. Antibody-mediated encephalitis. *New England Journal of Medicine* (2018) 378:840–851. doi:10.1056/NEJMra1708712
2. Dalmau J, Rosenfeld MR. Autoimmune encephalitis update. *Neuro-Oncology* (2014) 16:771–778. doi:10.1093/neuonc/nou030
3. Nóbrega PR, Pitombeira MS, Mendes LS, Krueger MB, Santos CF, De Menezes Morais NM, et al. Clinical features and inflammatory markers in autoimmune encephalitis associated with antibodies against neuronal surface in Brazilian patients. *Frontiers in Neurology* (2019) 10:1–6. doi:10.3389/fneur.2019.00472
4. Graus F, Titulaer MJ, Balu R, Benseler S, Bien CG, Cellucci T, et al. A clinical approach to diagnosis of autoimmune encephalitis. *The Lancet Neurology* (2016) 15:391–404. doi:10.1016/S1474-4422(15)00401-9
5. Melamud LI, Fernández VC, Manin A, Villa AM. Autoimmune encephalitis and immune therapy: lessons from Argentina. *Acta Neurologica Belgica* (2020) 120:565–572. doi:10.1007/s13760-018-1013-x
6. Da Silva-Júnior FP, Castro LHM, Andrade JQ, Bastos CG, Moreira CH, Valério RMF, et al. Serial and prolonged EEG monitoring in anti-N-Methyl-d-Aspartate receptor encephalitis. *Clinical Neurophysiology* (2014) 125:1541–1544. doi:10.1016/j.clinph.2014.01.001
7. Chavez-Castillo M, Ruiz-Garcia M, Herrera-Mora P. Characterization and Outcomes of Epileptic Seizures in Mexican Pediatric Patients With Anti-N-Methyl-D-Aspartate Receptor Encephalitis. *Cureus* (2020) 12:e8211. doi:10.7759/cureus.8211
8. Danieli D, Moraes ACM, Alves MP, Dutra LA, Höftberger R, Barsottini OGP, et al. Anti-N-methyl-D-aspartate receptor encephalitis and Epstein-Barr virus: another tale on autoimmunity? *European Journal of Neurology* (2017) 24:e46–e47. doi:10.1111/ene.13332
9. Pérez E, Ruggieri V, Monges S, Loos M, Caraballo R, Yerga A, et al. Antibody-mediated acute encephalitis against the ionotropic glutamate receptor activated by N-methyl-D-aspartate (NMDAR): analysis of pediatric cases in Argentina (Premio Benito Yelín) [Acute encephalitis anti-ionotropic glutamate receptor activated N-methyl-D -aspartate (NMDAR): analysis of eleven pediatric cases in Argentina (Benito Yelín Award)]. *Medicina (Buenos Aires)* (2013) 73 Suppl 1:1-9
10. Solís N, Salazar L, Hasbun R. Anti-NMDA Receptor antibody encephalitis with concomitant detection of Varicella zoster virus. *Journal of Clinical Virology* (2016) 83:26–28. doi:10.1016/j.jcv.2016.08.292
11. Erazo R, González J, Quintanilla C, Devaud C, Gayoso C, Toledo X, et al. Encefalitis subaguda por anticuerpos anti receptor de N-metil-D-aspartato. Serie de 13 casos pediátricos [Subacute anti-N-methyl-D-aspartate receptor encephalitis. A serie of 13 paediatric cases]. *Revista Chilena de Pediatria* (2016) 87:487–493. doi:10.1016/j.rchipe.2016.06.006
12. Nóbrega PR, Morais NMM, Braga-Neto P, Barros LSS, Honório FPP, Dellavance A, Hoftberger R, Dutra LA. NMDAR Encephalitis Associated With Acute Chikungunya Virus Infection: A New Trigger? *Frontiers in Pediatrics* (2020) 8:176. doi:10.3389/fped.2020.00176
13. Vale TC, Pedroso JL, Alquéres RA, Dutra LA, Barsottini OGP. Spontaneous downbeat nystagmus as a clue for the diagnosis of ataxia associated with anti-GAD antibodies. *Journal of the Neurological Sciences* (2015) 359:21–23. doi:10.1016/j.jns.2015.10.024
14. Pablo González R, Lorena Hudson A, Esteban Basáez M, Marcelo Miranda C. Encefalitis autoinmune por anticuerpos contra el receptor GABA-A: Caso clínico [Autoimmune encephalitis induced by antibodies against GABA-A receptor]. Caso clínico. *Revista Medica de Chile* (2016) 144:1491–1493. doi:10.4067/S0034-98872016001100017
15. Guevara C, Farias G, Silva-Rosas C, Alarcon P, Abudinen G, Espinoza J, et al. Encephalitis associated to metabotropic glutamate receptor 5 (mGluR5) antibodies in cerebrospinal fluid. *Frontiers in Immunology* (2018) 9: doi:10.3389/fimmu.2018.02568
16. Chaumont H, Petit A, Mameri T, Schollhammer R, Honnorat J, Lannuzel A. Successful Management of Anti-mGluR1 Encephalitis with Immunosuppressive Treatment: Dengue Virus as a Trigger? *Movement Disorders Clinical Practice* (2019) 6:727–728. doi:10.1002/mdc3.12841
17. Dubey D, Pittock SJ, Kelly CR, McKeon A, Lopez-Chiriboga AS, Lennon VA, et al. Autoimmune encephalitis epidemiology and a comparison to infectious encephalitis. *Annals of Neurology* (2018) 83:166–177. doi:10.1002/ana.25131
18. Titulaer MJ, McCracken L, Gabilondo I, Armangué T, Glaser C, Iizuka T, et al. Treatment and prognostic factors for long-term outcome in patients with anti-N-Methyl-D-Aspartate (NMDA) receptor encephalitis: a cohort study. *Lancet Neurology* (2013) 12:157–165. doi:10.1016/S1474-4422(12)70310-1
19. Dalmau J, Armangué T, Planagumà J, Radosevic M, Mannara F, Leypoldt F, et al. An update on anti-NMDA receptor encephalitis for neurologists and psychiatrists: mechanisms and models. *The Lancet Neurology* (2019) 18:1045–1057. doi:10.1016/S1474-4422(19)30244-3
20. do Valle DA, Galeazzi JSP, Machado MR, dos Santos VCSAR, da Silva AF, Lohr Júnior A, et al. Clinical variability of children with anti-N-methyl-d-aspartate receptor encephalitis in southern Brazil: a cases series and review of the literature. *Neurological Sciences* (2019) 40:351–356. doi:10.1007/s10072-018-3648-z
21. Bravo-Oro A, Acosta-Yebra D, Grimaldo-Zapata IP, Reyes-Vaca G. Atrofia cortical reversible secundaria a encefalitis por anticuerpos antirreceptor de NMDA. *Revista de Neurologia* (2015) 60:447–452. doi:10.33588/rn.6010.2014426
22. González-Latapi P, Rodríguez-Violante M, Cervantes-Arriaga A, Calleja-Castillo JM, González-Aguilar A. Encefalitis por anticuerpos antirreceptor de N-metil-D-aspartato (anti-NMDAR): reporte de un caso. *Gaceta Médica de México* (2014) 150:348–51.
23. Bravo-Oro A, Abud-Mendoza C, Quezada-Corona A, Dalmau J, Campos-Guevara V. Encefalitis por anticuerpos contra el receptor de NMDA: Experiencia con seis pacientes pediátricos. potencial eficacia del metotrexato. *Revista de Neurologia* (2013) 57:405–410. doi:10.33588/rn.5709.2013180
24. Borlot F, Santos MLF, Bandeira M, Liberalesso PB, Kok F, Löhr A, et al. Anti-N-methyl D-aspartate receptor encephalitis in childhood. *Jornal de Pediatria* (2012) 88:275–278. doi:10.2223/JPED.2172
25. Benjumea-Cuartas V, Eisermann M, Simonnet H, Hully M, Nabbout R, Desguerre I, et al. Unilateral predominance of abnormal movements: A characteristic feature of the pediatric anti-NMDA receptor encephalitis? *Epilepsy and Behavior Case Reports* (2017) 7:42–44. doi:10.1016/j.ebcr.2016.12.002
26. Gómez Castro JF, Salazar O, Conde Z. Índice de sospecha: manifestaciones psiquiátricas de la encefalitis por anticuerpos anti-NMDAR en pacientes pediátricos [Suspicion Index: Psychiatric Manifestations of NMDAR Encephalitis in Paediatric Patients]. *Revista Colombiana de Psiquiatría* (2017) 46:252–256. doi:10.1016/j.rcp.2016.10.002
27. Passareli V, Medeiros L, Simabukuro M, Baldocci M, Brucki S, Rocha MSG. arly occurrence of brief potentially ictal rhythmic discharges [B(I)RDs], and subsequent emergence of asymmetric extreme delta brush (EDB) in Anti-NMDA receptors encephalitis. *Arquivos de Neuro-Psiquiatria* (2016) 74:177. doi:10.1590/0004-282X20150176
28. Rangel-Guerra R, Camara-Lemarroy CR, Garcia-Arellano G, Rodriguez-Martinez AC, Galarza-Delgado DA. Could coenzyme Q10 supplementation have a role in the treatment of anti-NMDA receptor encephalitis? *Acta Neurologica Belgica* (2015) 115:85–86. doi:10.1007/s13760-014-0299-6
29. Schmitt SE, Pargeon K, Frechette ES, Hirsch LJ, Dalmau J, Friedman D. Extreme delta brush receptor encephalitis. *Neurology* (2012) 79:1094–1100. doi:10.1212/WNL.0b013e3182698cd8
30. Bastiaansen AEM, Van Sonderen A, Titulaer MJ. Autoimmune encephalitis with anti-leucine-rich glioma-inactivated 1 or anti-contactin-associated protein-like 2 antibodies (formerly called voltagegated potassium channel-complex antibodies). *Current Opinion in Neurology* (2017) 30:302–309. doi:10.1097/WCO.0000000000000444
31. González Otárula KA, Ugarnes G, Fernández Suárez M, D’giano C. Crisis distónicas faciobraquiales. Diagnóstico semiológico en la encefalitis límbica [Faciobrachial dystonic seizures. Semiologic diagnosis in limbic encephalitis]. *Medicina (Argentina)* (2015) 75:407–409.
32. Alvarez WT, Suarez JAM, Acosta JJM, Escalante CRI, Mercado AZE. Sporadic Creutzfeldt-Jakob disease mimic due to voltage gated potassium channel antibodies (VGKC) autoimmune encephalitis. (case report). *Journal of the Neurological Sciences* (2019) 405:91. doi:10.1016/j.jns.2019.10.942
33. Ibarra V, Jaureguiberry A, Moretta G, Torres C, Lazzarini G, Ceruzzi R, et al. Encefalitis límbica autoinmune. Neurologia Argentina. (2015) 7:112– Q18 6. doi: 10.1016/j.neuarg.2014.12.002
34. Uribe-San-Martín R, Ciampi E, Santibañez R, Irani SR, Márquez A, Cruz JP, et al. LGI1-antibody associated epilepsy successfully treated in the outpatient setting. Journal of Neuroimmunology. (2020) 345:577268. doi: 10.1016/j.jneuroim.2020.577268
35. Saraiva G, Santana de Lima G, Mendes Silva R, Bueno Carvalho A, de Castro Machado Y, Matos Pereira N, et al. Autoimmune encephalitis as differential diagnosis of temporal encephalitis. Journal of the Neurological Sciences. (2015) 357:e210. doi: 10.1016/j.jns.2015.08.721
36. Restrepo Martínez M, Bautista GP, Espínola-Nadurille M, Bayliss L. Red flags for suspecting anti-NMDAr encephalitis in a first psychotic episode: Report of two cases. Revista Colombiana de Psiquiatría (English ed). (2019) 48:127–130. doi: 10.1016/j.rcpeng.2017.10.003
37. Palomino-Lescano H, Segura-Chávez D, Quispe-Orozco D, Castro-Suarez S, Cruz WD la, Zapata-Luyo W, et al. Encefalitis autoinmune mediada por anticuerpos contra el receptor N-Metil-N-Aspartato: reporte de cuatro casos en Perú. Rev Peru Med Exp Salud Publica. (2019) 36:138– 44. doi: 10.17843/rpmesp.2019.361.3936
38. Nóbrega-Jr AW, Gregory CP, Schlindwein-Zanini R, Neves FS, Wolf P, Walz R, et al. Mesial temporal lobe epilepsy with hippocampal sclerosis is infrequently associated with neuronal autoantibodies. Epilepsia. (2018) 59:e152–e6. doi: 10.1111/epi.14534
39. Bustos J, Sánchez Y, Medina J, Olivieri R, Mojica J, Ortiz J. Autoinmune encephalitis associated to antibodies against the receptor N-methyl-D-aspartate: report of two cases. Biomédica. (2016) 37:20–5. doi: 10.7705/biomedica.v37i2.2909
40. Kerik-Rotenberg N, Diaz-Meneses I, Hernandez-Ramirez R, MuñozCasillas R, Reynoso-Mejia CA, Flores-Rivera J, et al. A Metabolic Brain Pattern Associated With Anti-N-Methyl-D-Aspartate Receptor Encephalitis. Psychosomatics. (2020) 61:39–48. doi: 10.1016/j.psym.2019.08.007
41. Restrepo-Martínez M, Ramírez-Bermúdez J, Bayliss L, Espinola-Nadurille M. Characterisation and outcome of neuropsychiatric symptoms in patients with anti-NMDAR encephalitis. Acta Neuropsychiatr. (2020) 32:92– 8. doi: 10.1017/neu.2019.46
42. Simabukuro MM, Nóbrega PR, Pitombeira M, Cavalcante WCP, Grativvol RS, Pinto LF, et al. The importance of recognizing faciobrachial dystonic seizures in rapidly progressive dementias. Dementia e Neuropsychologia. (2016) 10:351–57. doi: 10.1590/s1980-5764-2016dn1004016
43. Restrepo-Martínez M, Chacón-González J, Bayliss L, Ramírez-Bermúdez J, Fricchione GL, Espinola-Nadurille M. Delirious Mania as a Neuropsychiatric Presentation in Patients With Anti–N-methyl-D-aspartate Receptor Encephalitis. Psychosomatics. (2020) 61:64–9. doi: 10.1016/j.psym.2019.03.002
44. Reyna-Villasmil E, Mayner-Tresol G, Herrera-Moya P. Anti-N-methyl-Daspartate-receptor encephalitis due to ovarian teratoma. Medicina Clinica. (2017) 149:560–1. doi: 10.1016/j.medcli.2017.06.049
45. Cané VP, Lerchundi F, Sassul F, Consalvo DE, Amores M, Merovich M. Síndrome neuroléptico maligno asociado a encefalitis autoinmune por anticuerpos contra el receptor NMDA. Neurologia Argentina. (2019) 11:36– 9. doi: 10.1016/j.neuarg.2018.01.002
46. Martinez DB, Guerrero RT, Grandjean MB, Cartier LR. Encefalitis autoinmune reversible y anticuerpos anti-receptores de N-metil-Daspartato [Limbic encephalitis with positive anti-N-methyl-D-aspartate antibodies. Report of one case]. Revista medica de Chile. (2012) 140:1170–3. doi: 10.4067/S0034-98872012000900010
47. Jiménez-Zarazúa O, Martínez-Rivera MA, González-Carrillo PL, VélezRamírez LN, Alcocer-León M, Tafoya-Rojas SXL, et al. Headache, Delirium or Encephalitis? A Case of Residual Mutism Secondary to Anti-NMDA Receptor Encephalitis. Case Reports in Neurology. (2019) 11:330–43. doi: 10.1159/000504016
48. Mendes MFSG, Gomez RS, Pacheco JAS, Lima DVES, Santos TM, Téliz MAM, et al. Super-refractory status epilepticus due to anti-GAD-65 autoimmune encephalitis – biphasic presentations with discognitive crisis with generalization and posterior focal myoclonus. Journal of the Neurological Sciences. (2015) 357:e155. doi: 10.1016/j.jns.2015.08.523
49. Espinola-Nadurille M, Bustamante-Gomez P, Ramirez-Bermudez J, Bayliss L, Rivas-Alonso V, Flores-Rivera J. Frequency of neuropsychiatric disturbances in anti-NMDA receptor encephalitis. Acta Psychiatrica Scandinavica. (2018) 138:483–5. doi: 10.1111/acps.12963
50. Soares EMV, Kauark RBG, Rocha MSG, Brucki SMD. Anti-NMDA-R encephalitis: Follow-up of 24 months. Dement Neuropsychol. (2013) 7:304– 307. doi: 10.1590/S1980-57642013DN70300012
51. Solis JG, Olascoaga Lugo A, Rodríguez Florido MA, Sandoval Bonilla BA, Malagón Rangel J. Neurosarcoidosis Presentation as Adipsic Diabetes Insipidus Secondary to a Pituitary Stalk Lesion and Association with AntiNMDA Receptor Antibodies. Case Reports in Neurological Medicine. (2020) 2020:1–5. doi: 10.1155/2020/7956350
52. Espinola-Nadurille M, Flores-Rivera J, Rivas-Alonso V, Vargas-Cañas S, Fricchione GL, Bayliss L, et al. Catatonia in patients with anti-NMDA receptor encephalitis. Psychiatry and Clinical Neurosciences. (2019) 73:574– 80. doi: 10.1111/pcn.12867
53. Martinez HR, Olguin-Ramirez LA, Camara-Lemarroy CR. Lyme borreliosis as a trigger for NMDA receptor encephalitis? Neurological Sciences. (2018) 39:1815–7. doi: 10.1007/s10072-018-3497-9
54. Monteiro VL, Barreto FJN, Rocha PMB, Prado PHT do, Garcia FD, Correa H, Neves MCL das. Managing severe behavioral symptoms of a patient with anti-NMDAR encephalitis: case report and findings in current literature. Trends in Psychiatry and Psychotherapy. (2015) 37:47– 50. doi: 10.1590/2237-6089-2014-0036
55. Ramirez-Bermudez J, Restrepo-Martinez M, Diaz-Victoria AR, Espinola Nadurille M. Memantine as Adjuntive Therapy in a Patient With Anti-N-Methyl-D-Aspartate Receptor Encephalitis. Journal of clinical psychopharmacology. (2020) 40:92–3. doi: 10.1097/JCP.0000000000001145
56. Simabukuro MM, Watanabe RGS, Pinto LF, Guariglia C, Gonçalves DCME, Anghinah R. A successful case of anti-NMDAR encephalitis without tumor treated with a prolonged regimen of plasmapheresis. Dementia & Neuropsychologia. (2014) 8:87–9. doi: 10.1590/S1980-57642014DN81000014
57. Espinola-Nadurille M, Bautista-Gomez P, Flores J, Rivas-Alonso V, Perez-Esparza R, Solís-Vivanco R, et al. Non-inflammatory cerebrospinal fluid delays the diagnosis and start of immunotherapy in anti-NMDAR encephalitis. Arquivos de Neuro-Psiquiatria. (2018) 76:2–5. doi: 10.1590/0004-282x20170179
58. Bayliss L, Restrepo-Martínez M, Duarte A, Borja CC, Espinola-Nadurille M, Kahn DA. Are we missing subtle forms of anti-n-methyl-d-aspartate encephalitis with the current diagnostic approach? A case report. Journal of Psychiatric Practice. (2019) 25:383–90. doi: 10.1097/PRA.0000000000000412
59. Simabukuro MM, Freitas CHA, Castro LHM. A patient with a long history of relapsing psychosis and mania presenting with anti-NMDA receptor encephalitis ten years after first episode. Dementia e Neuropsychologia. (2015) 9:311–314. doi: 10.1590/1980-57642015dn93000016
60. Gómez Ávila FA, González-Aragón MF, Avellán ÁM, San-juan D. EEG Findings and Releases From Hospital for Patients With Anti-NMDA Receptor Encephalitis. Journal of Clinical Neurophysiology. (2020) Publish Ah:1–6. doi: 10.1097/WNP.0000000000000720
61. Delgado-García G, Cano-Nigenda V, Abundes-Corona A, Carrillo-Loza K, Calleja-Castillo J, Flores-Rivera J. Opisthotonus (Arc de cercle) in anti-NMDAR encephalitis. Arquivos de Neuro-Psiquiatria. (2018) 76:426. doi: 10.1590/0004-282x20180047
62. Alencar JMD, Ribeiro TAGJ, Diniz DS, Melo GDS, Bezerra IML, Oliveira Junior RMD, et al. Anti-GAD positive autoimmune encephalitis presenting with dementia, psychiatric symptoms and MS findings on the MRI: A case report. Journal of the Neurological Sciences. (2017) 381:536. doi: 10.1016/j.jns.2017.08.3720
63. Pérez-Gilabert Y. Encefalitis Autoinmune Antirreceptor de NMDA: Reporte de un Caso y Revisión de la Literatura. Revista Ecuatoriana de Neurologia. (2017) 26:46–52.
64. Lozano-Cuervo R, Espinola-Nadurille M, Restrepo-Martinez M, Rotenberg NK, Pollak TA, Ramirez-Bermudez J. Capgras delusion in anti-NMDAR encephalitis: A case of autoimmune psychosis. Asian Journal of Psychiatry. (2020) 54: doi: 10.1016/j.ajp.2020.102208
65. Monnerat BZ, Velasco TR, Nakano FN, Veriano A, Martins APP, Sakamoto AC. Opercular myoclonic-anarthric status epilepticus due to glutamic acid decarboxylase antibody-associated encephalitis. Epileptic Disorders. (2013) 15:342–346. doi: 10.1684/epd.2013.0596
66. da Silva AV, Neves MAPLF, Parizotto C, Filho OBR, Koltermann T. Mild encephalitis with reversible splenial lesion and anti-NMDA receptor encephalitis. Journal of the Neurological Sciences. (2015) 357:e213– e4. doi: 10.1016/j.jns.2015.08.732
